# Zebrafish as an experimental model for the simulation of neurological and craniofacial disorders

**DOI:** 10.14202/vetworld.2022.22-29

**Published:** 2022-01-11

**Authors:** Ashwin Rohan Rai, Teresa Joy, K. S. Rashmi, Rajalakshmi Rai, N. A. Vinodini, P. J. Jiji

**Affiliations:** 1 Department of Anatomy, Kasturba Medical College, Mangalore, Manipal Academy of Higher Education, Manipal, Karnataka, India; 2 Department of Anatomy, American University of Antigua College of Medicine, University Park, Coolidge, St. John’s, Antigua; 3 Department of Physiology, Kasturba Medical College, Mangalore, Manipal Academy of Higher Education, Manipal, Karnataka, India.

**Keywords:** craniofacial deformity, experimental model, neurological disorders, zebrafish

## Abstract

Zebrafish have gained momentum as a leading experimental model in recent years. At present, the zebrafish vertebrate model is increasingly used due to its multifactorial similarities to humans that include genetic, organ, and cellular factors. With the emergence of novel research techniques that are very expensive, it is necessary to develop affordable and valid experimental models. This review aimed to highlight some of the most important similarities between zebrafish and humans by emphasizing the relevance of the first in simulating neurological disorders and craniofacial deformity.

## Introduction

The zebrafish (*Danio rerio*; ZF) are freshwater fish belonging to the minnow tribe of the Cypriniformes order. It is a tropical fish native to Southeast Asia that is about 2.5-4 cm long. It is a popular aquarium fish, and it is easily available and cheap. ZF have the advantage of being able to tolerate water of low ion content [1,2]. They are shallow water habitants found in shallow ponds, canals, and streams, preferably in stagnant waters or slow flowing waters with a temperature between 6°C and 38°C [3]; hence, they can be well maintained in aquaria made of high-quality plastic and glass [4]. The capacity of the tank is determined as per the number of fish housed. A maximum of 10-12 ZF can be accommodated in 10 gal. of water. Soft water is ideal [5] for the housing of ZF, particularly when a pH of 7 is maintained [6]. ZF (being an omnivorous species) feed on animal and plant matter such as zooplankton, insects, algae, fish scales, sand, mud, and invertebrate eggs [7]. The laboratory ZF are fed with a commercially available tropical fish diet [8]. The sexual dimorphism in ZF is difficult to assess, but they can be distinguished by their body shape and color. Male ZF are more slender and display a golden red shade between their blue stripes, whereas female ZF exhibit a protruding belly, with their body having alternate blue stripes with silver stripes [9]. ZF undergo external fertilization and embryo development that produces a great number of progeny [10]. The optimal temperature for embryo production and breeding depends on the advent of light, on a 14:10 h light and dark cycle, on maintaining water temperature between 23°C and 28°C, and on maintaining water pH between 6.2 and 7.5 [11]. The mating processes involve three phases (namely, initiatory, receptive, and spawning phase) [12]. The female ZF release hundreds of eggs in a clutch at a time into the water [13]. They are highly fertile and lay offspring at quite an early age. The sexual maturity of the laboratory ZF is attained by the 3^rd^ month of their development, whereas their ideal reproductive age lies between 6 months and 1 year, while their lifespan can reach up to 5 years [9]. From the 3^rd^ day of fertilization, ZF are eligible for larval experiments. In fact, the ZF teleost lives in conditions that can be easily replicated *ex situ*.

ZF are used by many laboratories to substitute and/or to supplement higher vertebrate models due to their similarities regarding many features of their embryonic development and adult anatomy with those of humans [14,15]. Their relatively small size and radiant feature allow for the observation of the entire animal [16]; hence, ZF are an excellent animal model for learning the different pathophysiological processes in organogenesis, embryogenesis, and carcinogenesis as well as for undertaking pharmacological and toxicological studies [17-19]. Their egg size is small, and their translucent embryos make experimental manipulations such as cell labeling, lineage tracing, and cell transplantation significantly smoother. Unlike higher organisms, the ZF organ system is highly minimalistic [10]. The ZF model has benefited from the advances in genetics, as ZF tumor cell lines of any kind have been established by inducing the related gene expression and being able to effectively simulate cancer morphology and signaling pathways [20,21]. ZF models enable faster and relevant *in vivo* screening through imaging of the pathogenesis, thus providing critical insights into the molecular mechanisms of the disease [22,23]. The fully mapped ZF genome has revealed extensive homology with the human genome, thus making ZF an excellent tool for studying human pathologies [24]. In fact, ZF exhibit 70% gene homology with humans, while 84% of the genes identified in human diseases are also expressed in this teleost species [25].

The currently available animal models can simulate most of the cognitive and molecular aspects of human pathologies, but they are time constraint and expensive; in contrast, the ZF model is an efficient, robust, and unique drug screening tool [26]. Moreover, the ZF ventral telencephalon is homologous to the human striatum [27]. The ZF immune system is functionally developed within 48 h post-fertilization and, in a similar extent to that of mammals, it has neutrophils, natural killer cells, monocytes/macrophages, and non-specific cytotoxic cells [28]. There is continuous tooth regeneration in ZF, providing us with the opportunity to better understand tooth replacement in mammals. Even if they are more favorable as models than rodents in many research areas, ZF lacks skin, mammary glands, lungs, and prostate organs. However, like vertebrates, ZF have organs and tissues that show equivalent morphology and function to humans (heart, kidney, liver, pancreas, gastrointestinal tract, and brain) [24]. Studies have also reported that the sleep pattern in the brain of ZF is similar to that of sleeping humans, which involve rapid eye movement (REM) and non-REM sleep [29]. Jeong *et al*. [30] have revealed that the endothelial tight junction-based blood-brain barrier (BBB) of ZF is similar to that of humans. This BBB and blood-retinal barrier develop in ZF embryos by3 days post-infection on a transgenic ZF model has been revealed [31]. This review focuses on ZF as a novel model for studying neurodegenerative diseases and craniofacial development.

## ZF as Model for the Experimental Simulation of Neurological Disorders

The sequencing of the entire ZF genome has revealed that 70% of the proteins encoded by human genes are related to genes found in ZF, while 84% of the ZF genes are known to be associated with human diseases [32]. The nervous system of the ZF is simple, with individual elements (glutamatergic excitatory neurons and GABAergic inhibitory neurons) [33]. Decui *et al*. [34] have recently presented data that reveal that micronized resveratrol can prevent pentylenetetrazole (PTZ)-induced seizure in epilepsy, whereas results by Mazumder *et al*. [35] have suggested that the anticonvulsant action of the phosphatidylinositol-3-kinase inhibitor LY294002 can be highly potential against PTZ-induced convulsions. Choo *et al*. [36] have reported that newer antiepileptic drugs can trigger cognitive deficits in a PTZ-induced ZF model of epilepsy. It has been demonstrated that *Orthosiphon stamineus*, a leaf extract, has anticonvulsant properties and can affect the levels of tumor necrosis factor-alpha (TNF-α) and the occurrence of seizures in a ZF model of epilepsy [37]. Li *et al*. [38] have evaluated the hazardous effects of nano-silica and reserpine on ZF, claiming that both can cause depression, anxiety-like behavior, disturbed swimming, and exploratory behavior, along with reduced locomotion and a depressive phenotype. Furthermore, Li *et al*. [39] have assessed the neurotoxic effects of silica nanoparticles as a potential source of neurobehavioral toxicity in Parkinson’s disease (PD). On the other hand, Cronin and Grealy have developed a ZF model to screen drugs with potential neuroprotective and neurorestorative effects against PD [40]. Flinn *et al*, [41] have created a ZF model to investigate the mechanisms underlying neuronal cell death in early onset PD. Exposure to neurotoxins has been shown to cause a decrease in dopamine levels in ZF, thus making it an ideal teleost for the simulation and study of neuropsychiatric diseases, as underlined by Panula *et al*. [42].

Piato *et al*. [43] have developed a ZF model allowing the study of the effects of chronic stress (lasting 7-14 days) through the assessment of psychological and behavioral responses. Similarly, Chakravarty *et al*. [44] have managed to induce anxiety and mood-related disorders through the application of unpredictable chronic stress in ZF, and observed decreased neurogenesis complicated by mitochondrial dysfunction. Rambo *et al*. [45] have demonstrated that unpredictable chronic stress can increase locomotion in female ZF, whereas male ZF exhibited higher cortisol levels along with increased aggressive behavior. Demin *et al*. [46] demonstrated that the ZF tail immobilization test can act as an appropriate tool for the assessment of stress. According to Wei *et al*. [47], long-term exposure to bisphenol S can lead to stress and anxiety-like behavior in ZF. These symptoms were shown to be alleviated by tryptophan and fluoxetine treatment in a study undertaken by Giacomini *et al*. [48]. Sarasamma *et al*. [49] were able to demonstrate that exposure to zinc chloride can induce an impaired short-term memory and diminished locomotion that mimicked Alzheimer’s disease (AD), along with an induction of beta-amyloid (b-amyloid) and tau protein in the ZF brain. Koehler and Williams [26] have conducted a similar study in which a ZF AD model was generated using a protein phosphatase 2A inhibitor (okadaic acid); in their study, a treatment with lanthionine ketimine-5-ethyl ester has shown neuroprotective effects [26]. Moreover, Javed *et al*. [50] have demonstrated the potency of casein-coated gold nanoparticles in reversing the cognitive and locomotor dysfunction of ZF exposed to the toxicity of b-amyloid in AD. Richetti *et al*. [51] have previously reported the potential protective effect of quercetin and rutin against scopolamine-induced memory impairment in a ZF AD model, whereas Yendapalli *et al*. [52] have evaluated the dual effects of *Brassica juncea* and *Cynodon dactylon* extracts on the cognitive performance of scopolamine-induced amnesic ZF.

Pullaguri *et al*. [53] have recently found that triclosan (an antimicrobial agent) can induce anxiety-like behavior in ZF; this behavior was linked to altered acetylcholinesterase (AChE) activity in the ZF brain and skeletal muscle, in addition to an observed downregulation of structural protein in the skeletal muscle. Nema and Bhargava [54] have reported that the ZF exhibit a frequent freezing behavior after exposure to cypermethrin (a pesticide) with no variations in their brain superoxide dismutase and AChE activities. Nery *et al*. [55] have injected b-amyloid 1-42 into the hindbrain ventricle of ZF embryos, resulting in increased tau phosphorylation and impaired cognition that were reversed by lithium. Prolonged exposure to low doses of benzo(a)pyrene (an environmental hazard) has been reported to lead to the development of neurodegenerative diseases (such as PD and AD) in ZF [56], along with locomotor dysfunction [57]. Prenatal exposure to the water-soluble fraction of crude oil and lead has been shown to cause autism-like behavioral deficits in ZF larvae [58].

Moreover, when ZF larvae were exposed to valproic acid, they developed autism spectrum-like symptoms [59,60]. It can be safely argued that ZF are an ideal model for the simulation of deficits that are relevant to autism [61]. A study conducted by Gawel *et al*. [62] have proved that ZF should also be considered an emerging model for schizophrenia [62]. Finally, using ZF as a model, Barnhill *et al*. [63] have provided new insights into the pathogenesis of PD that can also be extended into mammalian models [63].

The number of ZF utilizing neuroscientific studies is increasing exponentially, and it is much exceeding that of rodents or other model species. Despite this, there is still much resistance regarding using ZF as a primary experimental model system. New potential for understanding the pathobiology of diverse central nervous system (CNS) impairments continues to emerge from studies focusing on the array of the ZFneuronal and behavioral capabilities. Although some of these models (such as those for anxiety, depression, and addiction) are well established in ZF, others (such as those for autism and obsessive-compulsive disorders) are not as well established. However, accumulating evidence suggests that a wide range of CNS disorders could be reliably simulated with the use of ZF [64].

Another common misunderstanding is that the ZF are exclusively useful for the undertaking of genetic and developmental studies. The expanding applicability of ZF models in practically every aspect of biomedicine, including the modeling of brain diseases [65-67], supports the opposite, as evidenced by the mounting evidence provided herein. Similarly, research using larval ZF has dominated the field for decades. This scenario has only recently begun to improve, as more and more laboratories recognize the significance of adult ZF models for studying brain illnesses that mimic human pathologies [65].

## ZF as a Model for the Study of Craniofacial Development

**Figure-1:**
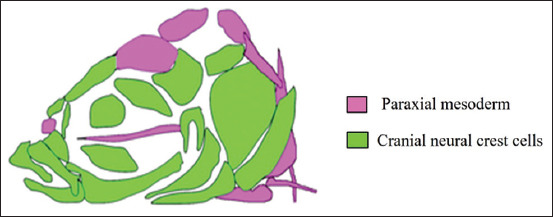
Craniofacial mesenchyme [72].

**Figure-2:**
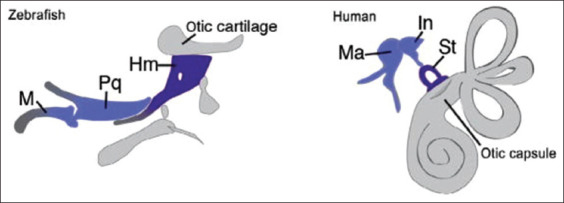
Zebrafish jaw homologous to mammalian middle ear [73].

**Figure-3:**
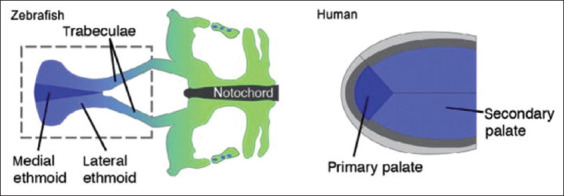
Zebrafish anterior neurocranium homologous to mammalian palate [73].

The ZF are a promising model for studying both the genetic and the environmental interactions occurring in craniofacial malformations such as palatal clefts. Over the last decade, the number of ZF genetic models for human disease-causing genes, such as those causing the cleft lip/palate, has exploded [68,69]. The ZF are a common model for visualizing the growth of the skull vault and of the cranial sutures. Kanther *et al*. [70] have hypothesized that the frontal and parietal bones of the skull meet and initiate suture formation after 30 days of development. Thyroid hormones are known to play an important role in the morphogenesis and ossification of the entire skeleton of ZF, according to a report by Keer *et al*. [71]. Like that of humans, the craniofacial mesenchyme is made up of migratory neural crest cells and paraxial mesoderm (Figure-1) [72]. The palatoquadrate and the posterior end of Meckel’s cage are evolutionarily related to the malleus and the incus of the mammalian middle ear. In ZF, the hyomandibula is the counterpart of the mammalian stapes (inner ear). The attachment of the stapes to the oval window in the otic capsule in humans is represented by the relationship between the hyomandibula and the otic cartilage in ZF. The ZF neurocranium anterior end is similar to the mammalian hard palate (Figures-2 and 3) [73]. The specialized diphyodont and heterodont dentition of humans has derived from the polyphyodonty homodont dentition of teleosts [15]. The ZF dentition has a rich vasculature for the processing of tooth development and replacement [74]. Based on these similarities, researchers have used ZF to investigate the phenotypic variability of human birth defects. A ZF study has found that combined exposure to platelet-derived growth factor receptor alpha and ethanol can cause craniofacial defects similar to those observed in fetal alcohol spectrum disorders (FASDs) [75]. According to a review conducted by Ellis *et al*. [76], exposure to the total particular matter (TPM) of cigarette smoke can induce craniofacial malformations in ZF when the TPM is >6 g/mL. Moreover, exposure to TPM has been shown to increase larval motility, to cause gross morphological deformities, and to distort the AhR pathway-associated enzyme activity. TPM toxicity is known to be influenced in part by genetic factors that regulate xenobiotic metabolism [76-78]. The Wnt/β-catenin signaling is a major pathway for craniofacial growth [79,80]. ZF exposed to alcohol during early gastrulation and neurulation have developed FASD symptoms that comprised differential expression of the sonic hedgehog pathway genes and the development of craniofacial malformations [81]. When ZF larvae were exposed to 100 mM (2.4%) of ethanol, they presented with a reduced ethmoid plate width that was then rescued by a low dose (1 nM) administration of exogenous retinoic acid (RA); moreover, when the RA was administered without a previous exposure of the ZF larvae to alcohol, the ZF developed a broader ethmoid plate [82].

A study conducted by Liu and Semina [83] who have demonstrated that a Paired-like homeodomain 2 transcription pathway deficiency can lead to abnormal ocular and craniofacial development. Treacher Collins syndrome, CHARGE syndrome [coloboma, heart defects, atresia choanae (also known as choanal atresia), growth retardation, genital abnormalities, and ear abnormalities], and Roberts syndrome have all been effectively standardized and can be simulated in ZF. These disorders comprise of cleft palate, microcephaly, smooth philtrum, and other malformities [75,84]. Exposure to dibutyl phthalate can induce defects in embryonic craniofacial development as reported by Jergensen *et al*. [85], whereas Cedron *et. al*. [86] has suggested that the overuse of acetaminophen or paracetamol can lead to the development of morphological abnormalities in craniofacialstructures. Chiquet *et al*. [87] have used ZF as a powerful tool for studying orofacial abnormalities, including that of the cleft lip/cleft palate. Küchler *et al*. [88] have used ZF embryos to assess the link between hypoxia and oral clefts. Cranial deformity accompanied by oxidative stress was observed in ZF embryos exposed to boscalid (a fungicide) in a study conducted by Wang *et al*. [89]. A study has been conducted to examine the effect of hormones on craniofacial malforma­tions on ZF larvae; During early chondrogenesis (1-2 dpf), ZF larvae exposed to exogenous 17β-estradiol (E2) have been shown to exhibit dose- and developmental stage-dependent craniofacial malformations with enhanced sensitivity [90]. With a focus on dental applicability, Makkar *et al*. [91] have studied the *in vivo* molecular toxicity profile of dental materials such as the mineral trioxide aggregate and biodentine using ZF models, whereas Rajendran *et al*. [92] have shown that zirconium oxide is more toxic to embryonic ZF even at low concentrations. The teeth in ZF larvae were treated with fluorine to examine them and compare the prevalence of dental fluorosis in primary and permanent teeth [93]. In an attempt to assess the protective effects of these products, Zhao *et al*. [94] have studied the impact of different metal alloy shells of Procelian fused metal alloy crowns on the growth of ZF embryos and larvae. Bisphenols have recently been the subject of a variety of ZF studies. For example, bisphenol A is an estrogen-like environmental compound that disrupts chondrocyte organization in the pharyngeal structures and induces apoptosis in ZF larvae, thus causing craniofacial malformations [95]. ZF are an excellent translational model for a wide range of craniofacial malformations, as well as for the assessment of the mechanisms of action associated with environmental pollutants and gene-environment interactions.

## Conclusion

A significant amount of craniofacial growth and neurodegenerative research has used the ZF as a model. The findings of these experiments have been included in this review as a proof of the value of ZF as an experimental setup that is cost-effective and easy to handle. Furthermore, the study of ZF will be particularly useful in the discovery and risk assessment of new teratogens and assisting in the translation of preclinical data into practice, thus informing our health advice to expecting mothers to reduce the risk of craniofacial malformations and developing neurological disorders. The latter should encourage new research to be conducted on the ZF model with the hope that it will prove beneficial for the human race.

## Authors’ Contributions

ARR and TJ: Contributed to the original draft, investigation, and editing of the manuscript. KSR and NAV: Collected the relevant literature and edited the manuscript. RR and PJJ: Revised the manuscript. All authors have read and approved the final manuscript.
